# Genome-wide association analyses for meat quality traits in Chinese Erhualian pigs and a Western Duroc × (Landrace × Yorkshire) commercial population

**DOI:** 10.1186/s12711-015-0120-x

**Published:** 2015-05-12

**Authors:** Xianxian Liu, Xinwei Xiong, Jie Yang, Lisheng Zhou, Bin Yang, Huashui Ai, Huanban Ma, Xianhua Xie, Yixuan Huang, Shaoming Fang, Shijun Xiao, Jun Ren, Junwu Ma, Lusheng Huang

**Affiliations:** Key Laboratory for Animal Biotechnology of Jiangxi Province and the Ministry of Agriculture of China, Jiangxi Agricultural University, Nanchang, 330045 China

## Abstract

**Background:**

Understanding the genetic mechanisms that underlie meat quality traits is essential to improve pork quality. To date, most quantitative trait loci (QTL) analyses have been performed on F_2_ crosses between outbred pig strains and have led to the identification of numerous QTL. However, because linkage disequilibrium is high in such crosses, QTL mapping precision is unsatisfactory and only a few QTL have been found to segregate within outbred strains, which limits their use to improve animal performance. To detect QTL in outbred pig populations of Chinese and Western origins, we performed genome-wide association studies (GWAS) for meat quality traits in Chinese purebred Erhualian pigs and a Western Duroc × (Landrace × Yorkshire) (DLY) commercial population.

**Methods:**

Three hundred and thirty six Chinese Erhualian and 610 DLY pigs were genotyped using the Illumina PorcineSNP60K Beadchip and evaluated for 20 meat quality traits. After quality control, 35 985 and 56 216 single nucleotide polymorphisms (SNPs) were available for the Chinese Erhualian and DLY datasets, respectively, and were used to perform two separate GWAS. We also performed a meta-analysis that combined *P*-values and effects of 29 516 SNPs that were common to Erhualian, DLY, F_2_ and Sutai pig populations.

**Results:**

We detected 28 and nine suggestive SNPs that surpassed the significance level for meat quality in Erhualian and DLY pigs, respectively. Among these SNPs, ss131261254 on pig chromosome 4 (SSC4) was the most significant (*P* = 7.97E-09) and was associated with drip loss in Erhualian pigs. Our results suggested that at least two QTL on SSC12 and on SSC15 may have pleiotropic effects on several related traits. All the QTL that were detected by GWAS were population-specific, including 12 novel regions. However, the meta-analysis revealed seven novel QTL for meat characteristics, which suggests the existence of common underlying variants that may differ in frequency across populations. These QTL regions contain several relevant candidate genes.

**Conclusions:**

These findings provide valuable insights into the molecular basis of convergent evolution of meat quality traits in Chinese and Western breeds that show divergent phenotypes. They may contribute to genetic improvement of purebreds for crossbred performance.

**Electronic supplementary material:**

The online version of this article (doi:10.1186/s12711-015-0120-x) contains supplementary material, which is available to authorized users.

## Background

Pork quality is an economically important trait in pig industry and has been one of the major objectives in pig breeding programs [1-3]. The main characteristics of meat include pH, drip loss, colour, firmness, moisture content, marbling and intramuscular fat content [4,5]. Obviously, all meat quality traits are complex traits that depend on multiple interacting factors, including genetic background, which may depend on species, major genes and gene interaction, and environmental background, which may depend on feeding, management and slaughter conditions [6]. A few meat quality traits can be predicted from analyses on small muscle biopsies sampled from living animals. However, to meet the requirements for testing multiple meat quality traits, it is necessary to conduct post-mortem sampling from animal carcasses, which increases the overall cost of measurement. Some meat quality traits may have negative and antagonistic relationships with other traits such as growth rate. In addition, some meat quality traits show a low to moderate heritability [7], which reduces the effectiveness of traditional phenotype-based breeding strategies for which estimation of breeding values relies only on the phenotypes of relatives. However, with the availability of pig whole-genome sequence data and the rapid development of molecular genetics, much progress has been made in untangling the molecular mechanisms that underlie meat quality traits. For example, it has been shown that the genes *insulin-like growth factor 2* (*IGF2*) [8,9], *melano-cortin 4 receptor* (*MC4R*) [9], *protein kinase, AMP-activated, gamma 3 non-catalytic subunit* (*PRKAG3*) [10] and *ryanodine receptor 1* (*RYR1*) [11] have major effects on meat production and quality in pigs. Identification of these genes has greatly benefited the pig industry [12].

Since 1994 when Andersson *et al.* [6] first published the genetic mapping of quantitative trait loci (QTL) in pigs, numerous QTL have been reported and deposited in the Pig QTL database (http://www.animalgenome.org/cgi-bin/QTLdb/SS/index). However, most of these QTL were identified through family-based linkage analyses using a limited number of microsatellites and they span large genomic regions i.e. with a size greater than15 cM, and considerable efforts are needed to refine the position of target QTL [13]. Recently, genome-wide association studies (GWAS) have emerged as a powerful new approach to identify QTL in livestock. By taking advantage of the high-throughput genotyping technologies (e.g. the Illumina Porcine60KSNP chip platform [14]) and of the much lower level of linkage disequilibrium (LD) in outbred populations than in resource families, GWAS has proven to be far more efficient to estimate location and effect of QTL than linkage analysis [15,16]. In addition, QTL linkage analyses are based on the assumption that alternative QTL alleles are fixed in each parental line of the experimental intercross. This is not the case for GWAS and the detected associations are based on the LD between SNPs and causative mutations [17].

To date, many porcine QTL have been mapped using crosses between outbred lines (e.g. Chinese breeds × Western breeds), but there are few studies on QTL analyses in outbred populations. In this study, we conducted GWAS for meat quality traits in Chinese Erhualian and Western DLY [Duroc × (Landrace × Yorkshire)] commercial populations. The Erhualian pig breed, like the Meishan breed, is a Chinese fat-type line that is well known for its high prolificacy, superior meat quality and strong resistance to harsh environments; thus, it is often included in Chinese pig breeding programs. Compared to the Erhualian breed, the DLY cross shows greater growth rate and meat productivity and currently has the biggest share of the pork market in China. Our aim was to map population-specific QTL at high resolution for meat quality traits in Chinese purebred Erhualian and western crossbred DLY populations.

Previously, we reported QTL scans using a White Duroc × Erhualian F_2_ resource population and Chinese Sutai pigs and the identification of a number of QTL that affect meat quality traits [18-24]. The Sutai pig is a synthetic line that derives from a cross between the Duroc breed (50%) and the Chinese Taihu (50%), which itself includes Erhualian, Meishan and Fengjing pigs [25]. It has been shown that the joint analysis of multiple phenotypes can increase the power of QTL detection, thus we performed a meta-analysis of GWAS on four experimental populations i.e. Erhualian, DLY, F_2_ and Sutai.

## Methods

### Ethics statement

All procedures involving animals followed the guidelines for the care and use of experimental animals approved by the State Council of the People’s Republic of China. The ethics committee of Jiangxi Agricultural University specifically approved this study.

### Animals and phenotypic traits

This study involved two populations: Chinese Erhualian pigs and Western DLY pigs. The Erhualian population comprised 168 barrows and 168 gilts, which were born and raised for two to three months on Erhualian cooperatives that were located around the Jiaoxi Changzhou city in Jiangsu province. They were then transferred to a farm in Nanchang city in two batches. The DLY population consisted of 306 males and 304 females, which were raised on a farm in Xiushui city. All experimental animals were fed on a similar diet under a standardized feeding and management regimen, and given free access to water, and then slaughtered at approximately 300 days of age for Erhualian pigs and 180 days for DLY pigs in the same commercial abattoir. The meta-analysis that combined four GWAS included two additional populations: F_2_ and Sutai pigs, as described by Ma *et al.* [26]. Briefly, 1029 F_2_ pigs that originated from nine F_1_ boars and 59 F_1_ sows, which were the progeny of two White Duroc sires and 17 Erhualian dams, were slaughtered at 240 ± 5 days of age and were submitted to standardized cutting of the carcass. The Sutai population consisted of 461 offspring from four Sutai boars and 55 Sutai sows that were also slaughtered at 240 ± 5 days of age. The growth rates of these four populations differed in the following order: growth rate of DLY > F2 and Sutai > Erhualian pigs. They were all fed to similar market weight (~90 kg) before slaughter and thus age at slaughter differed for each population.

Eight meat quality traits, including pH, EZ-drip loss, L* for lightness, a* for redness, b* for yellowness, moisture content, marbling and firmness were measured on the *longissimus* muscle (LM) between the 10th rib and the first lumbar vertebra and *semimembranosus* muscle (SM) from the left side of the carcass [1,19]. Meat characteristics of both LM and SM were measured for all Erhualian individuals, but for DLY individuals, only LM tissue samples were collected from carcasses due to the high cost of sampling. pH was measured twice on each sample using a Delta 320 pH meter and the average value of the two measurements was used in subsequent analyses. For Erhualian individuals, pH values and pH drop were measured at 45 min and 24 h after slaughter in the morning. However, all DLY pigs were slaughtered between 11 pm and 1 am. Based on measures from 30 DLY muscle samples, we observed that pH decreased within the first 24 hours post-mortem and was almost stabilized after 24 hours. Thus, to avoid night work, we measured pH for DLY individuals at 36 h post-mortem i.e. at 1 pm on the third day. Drip loss was assayed using an EZ-Drip Loss method [27-29]. Meat colour scores (ranging from 1 to 6, with 1 = pale and 6 = dark), marbling scores (ranging from 1 to 10 with 1 = devoid and 10 = overly abundant) and firmness scores (ranging from 1 to 5) were subjectively evaluated according to National Pork Producer Council (NPPC) guidelines [30]. Three colour parameters L*, a* and b* on the surface cuts of LM and SM were objectively evaluated with a CM-2600d/2500d Minolta Chroma meter. Moisture content was determined by the routine oven-drying method. Summary statistics for all traits investigated in the Erhualian and DLY populations are in Tables 1 and 2, respectively.

**Table 1:** **Descriptive statistics of meat quality traits of** ***longissimus*** **muscle (LM) and** ***semimembranosus*** **muscle (SM) from Chinese Erhualian pigs**

**Traits**	**N**	**Mean**	**SD** ^**a**^	**Min.**	**Max.**	**h** ^**2c**^
**pH** ^**b**^						
LM_pH45min	334	6.48	0.26	5.62	7.07	0.08
LM_pH24h	332	5.99	0.42	5.37	7.03	0.41
LM_pHdrop_45min-24 h	332	0.49	0.37	−0.10	1.29	0.16
SM_pH45min	334	6.42	0.28	5.66	7.13	0.07
SM_pH24h	334	6.12	0.38	5.34	7.00	0.33
SM_pHdrop_45min-24 h	334	0.31	0.35	−0.10	1.42	0.08
**Drip loss**						
LM_DripEZ_24h,%	333	1.14	1.14	0.18	6.13	0.12
SM_DripEZ_24h,%	331	0.87	0.80	0.18	6.49	0.05
**Meat colour measures**						
LM_ColorM_L24h	334	46.10	4.47	36.66	60.15	0.18
LM_ColorM_a24h	333	2.61	1.43	−0.28	7.38	0.12
LM_ColorM_b24h	334	5.63	1.83	1.29	13.09	0.32
LM_ColorScore_24h (1–6)	334	3.20	0.62	1.50	4.50	0.05
SM_ColorM_L24h	334	39.39	3.52	31.14	51.25	0.25
SM_ColorM_a24h	332	5.29	1.49	1.91	9.58	0.31
SM_ColorM_b24h	332	4.86	2.08	0.75	11.47	0.56
SM_ColorScore_24h (1–6)	334	4.09	0.54	2.0	5.5	0.35
**Moisture content**						
LM_MoistureContent, %	332	73.63	1.79	66.32	79.71	0.18
**Subjective scores**						
LM_Firmness (1–5)	334	2.17	0.51	1.0	3.5	0.36
LM_Marbling (1–10)	331	3.95	1.90	1.5	10.0	0.29
SM_Marbling (1–10)	334	2.16	0.58	1.5	5.0	0.05

^a^Standard deviation; ^b^pH was measured on samples of LM and SM 45 min and 24 h post-mortem; ^c^heritability estimates.

**Table 2:** **Descriptive statistics of meat quality traits of** ***longissimus*** **muscle (LM) from Western DLY pigs** ^**1**^

**Traits**	**N**	**Mean**	**S.D.**	**Min.**	**Max.**	**h** ^**2**^
**pH**						
LM_pH36h	580	5.50	0.25	5.04	6.42	0.34
**Drip loss**						
LM_DripEZ_36h, %	583	3.35	2.09	0.15	9.52	0.42
**Meat colour measures**						
LM_ColorM_L36h	584	47.03	3.31	34.58	58.52	0.37
LM_ColorM_a36h	584	1.51	1.11	−1.45	6.62	0.40
LM_ColorM_b36h	584	5.68	1.47	1.38	9.21	0.34
LM_ColorScore_36h(1–6)	584	3.05	0.81	1.0	4.5	0.35
**Moisture content**						
LM_MoistureContent, %	610	75.00	0.71	72.00	78.00	0.27
**Subjective scores**						
LM_Firmness (1–5)	575	2.86	0.73	1.0	5.0	0.19
LM_Marbling (1–10)	610	2.73	0.64	1.5	5.5	0.18

^1^See footnotes in Table 1.

### Genotyping and quality control

Genomic DNA was isolated from ear tissue using a standard phenol/chloroform method and dissolved in Tris-EDTA buffer. DNA quality and concentration were determined using a Nanodrop-1000 spectrophotometer (Thermo Fisher, USA). A total of 336 Chinese Erhualian and 610 DLY pigs were genotyped for 61 565 SNPs using the Illumina PorcineSNP60K Beadchip according to the manufacturer^’^s protocol. Quality control procedures were carried out to remove SNPs with a call rate greater than 90%, a minor allele frequency (MAF) less than 0.01 and a significant deviation from Hardy-Weinberg equilibrium (*P* ≤ 10^−5^); moreover, animals with more than 10% missing genotypes or more than 5% Mendelian errors were removed from the dataset. A final set of 35 985 SNPs for 331 Chinese Erhualian and 56 216 SNPs for 610 DLY pigs were used for further statistical analysis. The large difference in number of SNPs that were retained for each population is explained by the fact SNPs on the Illumina PorcineSNP60K Beadchip were primarily ascertained based on a small panel of western pig genomes and that more SNPs had a MAF less than 0.01 for the Chinese Erhualian dataset than for western DLY dataset. SNP chromosomal positions were based on the current pig genome assembly (*Sus Scrofa* Build 10.2 assembly).

### Statistical analyses

Heritability of meat quality traits was estimated using the *polygenic* function of GenABEL v1.7 [31]. Genome-wide association studies (GWAS) analyses were performed using *polygenic* and *mmscore* function of GenABEL v1.7 [31]. Associations between SNPs and phenotypic values were evaluated using a generalized linear mixed model [32,33], which accounted for population structure by fitting the covariance among individuals inferred from high-density SNP data. The variance-covariance matrix was proportional to genome-wide identity-by-state [34]. Sex and batch were fitted as fixed effects in the model. Bonferroni-corrected thresholds of 0.05/N and 1/N, where N is the number of SNPs used for the analyses, were adopted for the 5% genome-wide and suggestive significance, respectively [35,36]. After quality control, the genome-wide and suggestive significance thresholds were 1.38E-06 (0.05/35 985) and 2.77E-05 (1/35 985), respectively for the Chinese Erhualian dataset, and 8.89E-07 (0.05/56216) and 1.78E-05 (1/56216), respectively for the DLY dataset. In addition, a less stringent threshold of 1.00E-04 was applied in order to detect moderate associations and possible pleiotropic effects of QTL on correlated traits.

Since population stratification is generally considered as a major factor that influences GWAS results [37], it was assessed by examining the distribution of test statistics generated from the thousands of association tests and assessing their deviation from the null distribution (i.e. the distribution expected under the null hypothesis of no SNP associated with the trait) in quantile-quantile (Q-Q) plots [37]. The Q-Q plots were generated using R software.

A meta-analysis was performed using a Z-score approach in the METAL software [38] that combines *P*-values and effects of a set of 29 516 SNPs that were common to all four populations i.e. Erhualian, DLY, F_2_ and Sutai.

## Results

### Assessment of population stratification

In this study, we conducted two separate single-population GWAS i.e. on Erhualian and DLY populations and a meta-analysis of GWAS on four populations. The Q-Q plots of the test statistics for each separate GWAS and the meta-analysis are in Figure S1 [See Additional file 1: Figure S1] and Figure S2 [See Additional file 2: Figure S2], respectively. Average genomic inflation factors (λ) for each GWAS were equal to 1.01 and 1.04 for the Erhualian and DLY populations, respectively, and to 1.08 for the combined populations, which indicated the absence of any obvious population stratification.

### Results of the separate GWAS for the Erhualian and DLY populations

**Figure 1:**
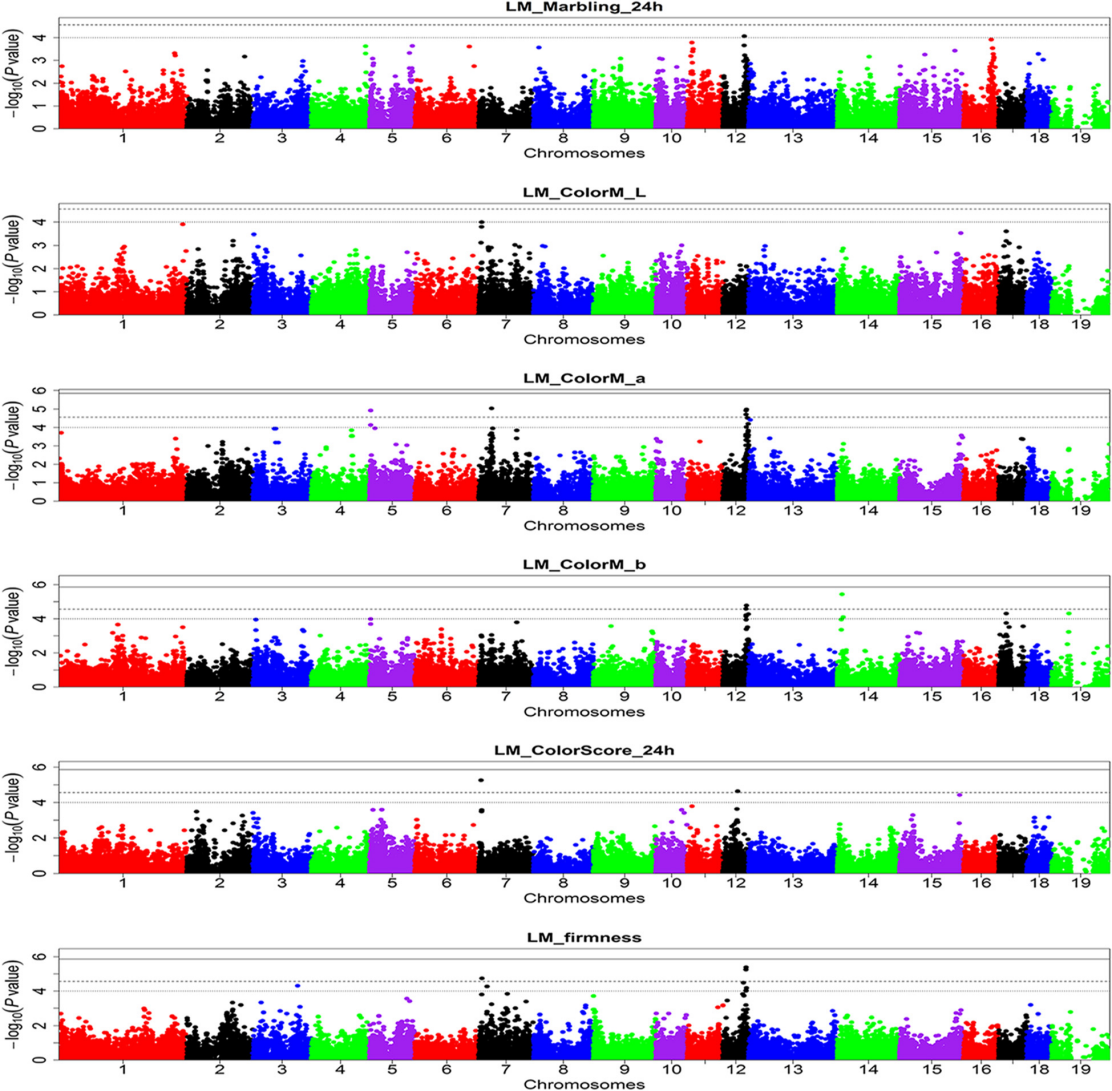
**Manhattans plots of the GWAS for meat quality traits in the Erhualian population.** In the Manhattan plots, negative log_10_ *P* values of the quantified SNPs were plotted against their genomic positions; SNPs on different chromosomes are indicated by different colours; dotted, dashed and solid lines correspond to the thresholds of 1.00E-04, 2.77E-05 and 1.38E-06, respectively.

Overall, we identified 37 SNPs (one unmapped SNP) on 14 chromosomes that were significantly associated with pH, drip loss, meat colour, moisture content and firmness in these populations (Table 3), which demonstrates the diverse genetic architecture of meat quality traits in pigs. Among these 37 associations, 28 were identified for the Erhualian population and nine for the DLY population; only one SNP (ss131261254 on SSC4 for LM_DripEZ_24h trait in the Erhualian dataset) exceeded the 5% genome-wide significance level (*P* = 7.97E-09). No SNP was associated with marbling at the GWAS significance level. Manhattan plots of GWAS for the 20 meat quality traits that were analyzed in the two populations are in Figure 1 and Figure S3 [See Additional file 3: Figure S3].

**Table 3:** **Description of SNPs significantly associated with meat quality traits in Erhualian and DLY pigs**

**Traits** ^**2**^	**Peak SNP**	**No.** ^**3**^	**Chr**	**Pos (bp)** ^**4**^	**PPL** ^**5**^	**Nearest genes** ^**6**^	**Freq** ^**7**^	**Effects** ^**8**^	***P*** **-value** ^**9**^
**EHL** ^**1**^									
LM_pH24h	ss131409440	1	9	144 399 17		*INTS7*	0.67	0.088	9.78E-06
SM_pH24h	ss131225834	1	3	15 271 92		*WBSCR17*	0.33	0.185	3.13E-06
LM_pHdrop_45min-24 h	ss107857062	1	16	79 490 30	c	*SEMA5A*	0.53	0.158	8.05E-06
LM_DripEZ_24h	ss131261254	1	4	44 336 60		*ENSSSCG00000019169*	0.15	0.686	7.97E-09
LM_DripEZ_24h	ss478940762	2	4	35 761 053		*RIMS2*	0.12	0.618	2.62E-06
LM_DripEZ_24h	ss131270350	1	4	85 151 430		*ST18*	0.14	1.092	6.26E-06
SM_DripEZ_24h	ss131134976	1	1	181 797 733		*MEGF11*	0.50	0.247	2.31E-06
LM_ColorM_a24h	ss131341943	1	7	31 500 144		*KLHL31*	0.60	0.505	9.1E-06
LM_ColorM_a24h	ss478937995	3	12	58 130 567	b	*MYH3*	0.18	0.945	1.04E-05
LM_ColorM_a24h	ss131565824	1	5	2 588 817		*EFCAB6*	0.65	0.545	1.18E-05
LM_ColorM_b24h	ss120027962	1	14	11 463 494		*BNIP3L*	0.02	1.628	3.64E-06
LM_ColorM_b24h	ss478937995	2	12	58 130 567	b	*MYH3*	0.18	0.976	1.65E-05
LM_ColorScore_24h	ss131352394	1	7	5 406 955	a	*EEF1E1*	0.74	0.234	5.49E-06
LM_ColorScore_24h	ss131461879	1	12	36 158 023		*SUPT4H1*	0.63	0.208	2.29E-05
SM_ColorM_b24h	ss107878019	1	12	9 939 154		*ENSSSCG00000025361*	0.95	1.435	1.88E-05
SM_ColorScore_24h	ss478943784	1	17	56 358 556		*SULF2*	0.33	0.235	7.70E-06
LM_MoistureContent	ss131186989	1	2	43 911 957		*LDHA*	0.01	1.010	5.18E-06
LM_MoistureContent	ss131458048	1	12	24 427 829		*NFE2L1*	0.01	0.860	5.31E-06
LM_MoistureContent	ss131054125	1	8	14 014 229		*ENSSSCG00000029791*	0.51	0.653	8.07E-06
LM_MoistureContent	ss131538960	2	16	74 982 852	c	*MFAP3*	0.86	1.112	1.67E-05
LM_Firmness	ss131096544	2	12	57 123 997	b	*WDR16*	0.72	0.232	4.10E-06
LM_Firmness	ss478937524	1	7	7 764 396	a	*TMEM14C*	0.64	0.225	1.81E-05
**DLY** ^**1**^									
LM_DripEZ_36h	ss131494523	1	13	141 530 003		*FAM43A*	0.36	0.435	1.53E-05
LM_DripEZ_36h	ss131400253	1	9	95 957 104	d	*SNX13*	0.88	0.740	1.57E-05
LM_ColorM_a36h	ss107833468	1	15	134 397 712	e	*RESP18*	0.37	0.245	1.78E-05
LM_ColorM_b36h	ss107856037	1	15	92 671 886		*AGPS*	0.32	0.569	9.17E-06
LM_ColorScore_36h	ss131398982	1	9	84 832 035	d	*ASNS*	0.62	0.122	4.78E-06
LM_ColorScore_36h	ss131528409	1	15	127 419 860	e	*IKZF2*	0.56	0.153	7.27E-06
LM_ColorScore_36h	ss107897208	1	9	45 577 347		*NCAM1*	0.44	0.148	9.96E-06
LM_ColorScore_36h	ss107799219	1	0	0			0.98	0.543	1.46E-05
LM_Firmness	ss131184920	1	2	34 977 540		*KIF18A*	0.37	0.172	1.47E-05

^1^Chinese Erhualian indigenous population and Western Duroc × (Landrace × Yorkshire) commercial population; ^2^description of the traits is in Table 1; ^3^number of SNPs that surpassed the suggestive significance level within the QTL regions; ^4^positions of the most significant SNP according to the *Sus Scrofa* Build 10.2 assembly; ^5^possible pleiotropic loci (PPL) were indicated by common letters on different rows; ^6^annotated genes nearest to the most significant SNP; gene names starting with ENSSSCG follow the Ensembl nomenclature while other gene symbols follow the HUGO nomenclature; ^7,8^frequencies and effects of the allele that increases phenotype value in the two populations; ^9^genome-wide significant associations are underlined.

### pH values and drip loss

We identified three significant SNPs associated with pH values in the Erhualian dataset, including one for LM_pH24h, one for SM_pH24h and one for LM_pHdrop_45min-24 h, which were localized on SSC9 (SSC for *Sus scrofa* chromosome), SSC3 and SSC16, respectively. No significant SNP was associated with pH value in the DLY dataset. Five significant SNPs were associated with drip loss in the Erhualian dataset, including four SNPs for drip loss of LM on SSC4 and one SNP for drip loss of SM on SSC1. In the DLY dataset, two SNPs associated with drip loss of LM were identified on SSC13 and SSC9, respectively. No common QTL was found between the two populations.

### Meat colour

Twelve and six significant SNPs associated with meat colour parameters were identified in the Erhualian and DLY datasets, respectively. In the Erhualian dataset, the most significant SNP was ss131341943 on SSC7 for LM_ColorM_a24h; SNP ss478937995 at 58.13 Mb on SSC12 was associated with both LM_ColorM_a24h and LM_ColorM_b24h. In addition, SNP ss131461879 at 36.15 Mb on SSC12 and SNP ss131352394 on SSC7 were shown to have effects on LM_ColorScore_24h in this dataset. SNPs for SM_ColorM_b24h and SM_ColorScore_24h were identified on SSC12 and SSC17, respectively. We did not detect any common region for the same trait on LM and SM. However, except for one unmapped SNP, all SNPs that were associated with meat colour of LM in the DLY dataset were located on SSC9 and SSC15. SNP ss107833468 for LM_ColorM_a36h at 134.39 Mb on SSC15 is the most significant SNP and is located near SNP ss131528409 at 127.41 Mb for LM_ColorScore_36h. The other two SNPs for LM_ColorScore_36h were detected on SSC9.

### Moisture content and firmness scores

In the Erhualian dataset, five and three SNPs were significantly associated with LM_MoistureContent and LM_Firmness, respectively. All the SNPs associated with LM_MoistureContent are located in four QTL regions on SSC2, SSC8, SSC12 and SSC16. In the DLY dataset, no significant SNP was associated with LM_MoistureContent and only one SNP ss131184920 on SSC2 was detected for LM_Firmness.

### Loci detected by the meta-analysis of GWAS across four populations

Meta-analysis is a powerful method that can reveal otherwise hidden or unclear associations that are detected by independent studies [38]. Our meta-analysis of GWAS across four populations revealed 16 SNPs associated with different meat quality traits: four for LM_pH24h, one for LM_DripZ_24h, one for LM_ColorScore_24h, five for LM_MoistureContent, one for LM_ColorM_a24h, two for LM_ColorM_b24h and two for LM_ColorM_L24h (Table 4 and Figure S4 [See Additional file 4: Figure S4]). Among these 16 SNPs, the most significant association was observed for SNP ss131289803 on SSC5 for LM_MoistureContent (*P* = 2.52E-07).

**Table 4:** **Description of SNPs significantly associated with meat quality traits in the meta-analysis of four pig populations (Erhualian, DLY, F**_**2**_ **and Sutai)** ^**1**^

**Traits**	**Peak SNP**	**No.**	**Chr**	**Pos (bp)**	**Nearest genes**	***P*** **-value**
LM_pH24h	ss131233621	1	3	136 625 292	*ID2*	3.63E-06
LM_pH24h	ss107835446	2	7	115 457 433	*ENSSSCG00000018967*	9.39E-06
LM_pH24h	ss131101693	1	X	141 793 169	*HAUS7*	2.46E-05
LM_DripEZ_24h	ss131380751	1	8	143 349 693	*WDFY3*	1.20E-05
LM_ColorM_L24h	ss131499292	2	14	118 515 539	*PI4K2A*	1.91E-05
LM_ColorM_a24h	ss131529154	1	15	133 970 166	*CDK5R2*	5.62E-06
LM_ColorM_b24h	ss131233621	1	3	136 625 292	*ID2*	1.04E-05
LM_ColorM_b24h	ss107823551	1	7	94 809 941	*ENSSSCG00000002270*	2.12E-05
LM_ColorScore_24h	ss131508309	1	14	18 052 581	*ENSSSCG00000021805*	1.16E-05
LM_MoistureContent	ss131289803	1	5	68 572 410	*PARP11*	2.52E-07
LM_MoistureContent	ss107806758	4	7	35 177 641	*SPDEF*	5.87E-06

^1^See footnotes in Table 3.

## Discussion

### Comparison between the QTL identified in the current and previous studies

A large number of QTL have been identified in multiple F_2_ intercrosses between Chinese and Western outbred pig strains, but few QTL have been detected within the founder strains (particularly the Chinese indigenous pig breeds), which restricts the use of maker-assisted selection to improve the performance of the founder animals and that of the crossbred animals. Here, we analyzed replicability of GWAS results between intercrosses and Chinese- and Western-type outbred populations. In the Erhualian dataset, we found a significant SNP ss131225834 for SM_pH24h at 15.27 Mb on SSC3 (Table 3), which is close to a locus that was previously detected for the same trait in Sutai pigs [26]. In addition, three significant SNPs for colour a* of LM were identified in a region around 58.13 Mb on SSC12, which confirms the locus reported by Luo et al. [39] for a Large White × Minzhu F_2_ intercross population. Thus, our results highlight several QTL that segregate in both F_2_ intercrosses and Chinese purebred Erhualian pigs. The position of these QTL could be further refined since the extent of LD is smaller in Chinese breeds than in western breeds and intercrosses [40,41].

### Detection of novel QTL

Our GWAS results not only confirmed a number of previously reported QTL but also revealed 12 novel loci. Six of these 12 loci were detected using Erhualian pig data, including: ss131409440 on SSC9 for LM_pH24h, ss478943784 on SSC17 for SM_ColorScore_24h, ss131186989 on SSC2, ss131054125 on SSC8 and ss131538960 on SSC16 for LM_MoistureContent, ss131096544 on SSC12 for LM_Firmness. The remaining six loci were identified using DLY pig data, including ss131494523 on SSC13 and ss131400253 on SSC9 for LM_DripEZ_36h, ss107856037 on SSC15 for LM_ColorM_b36h, ss131398982 and ss107897208 on SSC9 for LM_ColorScore_36h and ss131184920 on SSC2 for LM_Firmness.

### Possible pleiotropic QTL

Our results indicate that some regions may have pleiotropic effects on different meat quality traits. Using the Erhualian pig data, a 2.36 Mb region (between 5.40 and 7.76 Mb) on SSC7 was shown to contain SNPs that are associated with three related traits, including the most significant SNP ss120019062 for LM_ColorM_L24h at 7.02 Mb (*P* = 1.01E-04), SNP ss131352394 for LM_ColorScore_24h at 5.41 Mb and SNP ss478937524 for LM_Firmness at 7.76 Mb (Figure 1 and Table 3). On SSC12, SNP ss478937995 at 58.13 Mb was associated both with LM_ColorM_a24h and LM_ColorM_b24h and is close to SNP ss131096544 at 57.12 Mb that is associated with LM_Firmness. Interestingly, the most significant SNP, ss131466676 for LM_Marbling_24h (*P* = 8.59E-05), was also identified at 52.72 Mb on SSC12 (Figure 1), although it did not reach the suggestive significance level. This relatively lower significance might be due to the lack of precision in phenotypic measurement, the frequency and the size of the effect of the allele, and the number of loci that affect the trait. Coincidently, Luo et al. [39] also reported several significant SNPs for intramuscular fat content (IMF), marbling and meat colour on SSC12 in the proximal region between 46.90 and 55.22 Mb. In addition, the most significant SNPs for pH drop and moisture content of LM in the Erhualian dataset were located within a region of approximately 4.51 Mb (between74.98 and 79.49 Mb) on SSC16 (Table 3 and Figure S3).

For the DLY pig data, a 6.98 Mb region (between 127.41 and 134.39 Mb) on SSC15 was associated with LM_ColorM_a36h and LM_ColorScore_36h. The correlation coefficient between drip loss and colour score of LM was negative and highly significant (r = −0.62, *P* < 10^−4^); two adjacent SNPs associated with both traits were within a region of 11.12 Mb (between 84.83 and 95.95 Mb) on SSC9, which indicates that this region may contain a pleiotropic QTL (Table 3). Since the Bonferroni-corrected thresholds applied in our study are very stringent, it is likely that some QTL are missed if their effects are not strong enough. For example, if this threshold is reduced to 10^−4^, a 1.98 Mb region (between 36.07 and 38.05 Mb) on SSC7 is detected that contains several SNPs associated with LM_DripEZ_36h (*P* = 1.01E-04), LM_ColorM_L36h (*P* = 2.25E-05) and LM_ColorScore_36h (*P* = 3.44E-05) for the DLY pig data [See Additional file 3: Figure S3].

### All loci identified by the GWAS are population-specific or tissue-specific

The results that we obtained for the separate GWAS on the Erhualian and DLY populations did not reveal any common loci between these two pig breeds (Table 3), which suggests that major genes that lead to the marked phenotypic variations within the Erhualian and the DLY populations are distinct. However, it cannot be excluded that the two populations share a few of the minor genes that affect these traits. Moreover, our results showed clear differences in the genetic loci that influence meat quality traits between LM and SM in Erhualian pigs, which suggest that these two muscle tissues have distinct metabolic characteristics and contractile properties.

### Impact of the difference in phenotype ascertainment across populations on the results of the meta-analysis

It has been suggested that a difference in the approaches used to dissect phenotypes across populations can reduce the power of statistical tests in a meta-analysis [42]. In our study, there was a certain phenotypic heterogeneity. First, DLY, F_2_, Sutai and Erhualian pigs were slaughtered at different ages, i.e. 180, 240, 240 and 300 days, respectively, which correspond to the different time lengths required for the animals to grow to market weight. We consider that it is appropriate to measure meat quality in pigs at market weight and that the phenotypic data from these populations is comparable and can be used for meta-analysis. For the DLY pigs, due to practical difficulties, we conducted meat quality measurements at 36 h post-mortem while they were performed at 24 h post-mortem for the other pig populations. However, we found that phenotypic values at 24 h and 36 h post-mortem were approximately the same and thus, the level of phenotypic heterogeneity observed in this study is probably low.

Furthermore, unlike the direct analysis of pooled individual-level data, a meta-analysis uses the summary statistics from individual studies as the data points. Here, we applied a Z-score approach that combined P-values and the direction of effects observed in each population. This method can greatly alleviate the interference of different design factors from individual studies. Therefore, there may be an effect due to the different protocols used to dissect phenotypes among the test populations on the results of meta-analysis, but it is probably limited.

### Comparison of the loci identified by the meta-analysis with those by the single-population GWAS

The meta-analysis involved four populations: Erhualian, DLY hybrid, F_2_ (White Duroc × Erhualian) and Sutai (Duroc × Taihu) pigs. Both F_2_ and Sutai pigs share some genetic background with Duroc and Erhualian pigs, thus these populations may harbour some common QTL alleles. Moreover, meta-analysis is a tool for aggregating information from multiple independent studies so that it can provide more power to identify associated variants with small effect sizes or low frequencies. Therefore, this meta-analysis enabled us to identify several novel loci that were not detected by separate single-population GWAS. In addition to the SNP on SSCX that was detected using the F_2_ pig data, three additional SNPs for LM_pH24h on SSC3 and SSC7 were only detected by the meta-analysis. SNP ss131380751 on SSC8 for LM_DripEZ, SNP ss131499292 on SSC14 for LM_ColorScore_24h, SNP ss131289803 on SSC5 for LM_MoistureContent and SNP ss131233621 on SSC3 for LM_ColorM_b24h were also only detected by the meta-analysis. The most significant SNP ss131233621 on SSC3 was the first SNP found to be associated with both LM_ColorM_b24h and LM_pH24h. Compared to independent analyses, the joint analysis enabled us to obtain more precise locations and stronger significance for some QTL. For example, the most significant SNP ss131529154 for LM_ColorM_a24h that was revealed by the meta-analysis (Table 4), was located at 133.97 Mb on SSC15, which was closer to the known causative gene *PRKAG3* at 133.80 Mb, than the most significant SNP ss107833468 (at 134.39 Mb) identified by the GWAS on the DLY population only. The *P*-value for the locus for LM_ColorM_a24h on SSC15 was smaller (*P* = 5.62E-06) in the combined analysis than in the analysis with the DLY (*P* = 1.78E-05) population only (Table 3). None of the single-population GWAS detected significant SNPs for LM_ColorM_L24h; however, two significant SNPs on SSC14 were detected by the meta-analysis ([See Additional file 4: Figure S4] and Table 4). These results demonstrate that meta-analysis of GWAS has additional power to identify common variants across populations.

It should be noted that, in general, allelic effect direction of the above-mentioned SNPs that showed a higher significance in the meta-analysis was consistent across all tested populations. In contrast, the level of significance of SNP ss478940762 for drip loss on SSC4 decreased in the meta-analysis (5.89E-05) compared to the GWAS using only the Erhualian population (2.62E-06), which may be caused by opposite allelic directions of this SNP in different populations.

### Plausible candidate genes at the identified loci

We searched for promising candidate genes with functional relevance to the studied traits in an interval of 1 Mb centred on the most significant SNP at each significant locus. Based on the Erhualian dataset, the *PPP2R5A* gene located at about 270 kb upstream of the most significant SNP (ss131409440) is the best candidate gene for LM_pH24h. This gene encodes a protein that belongs to the phosphatase 2A regulatory subunit B family and is highly expressed in heart and skeletal muscle [43]. *PPP2R5A* is involved in the negative control of cell growth and division and plays a role in the Bio Systems Pathways that are directly involved in glycogen metabolism.

As suggested by Luo et al. [39], *MYH3*, a member of the *MYH* family, which contains the significant SNP ss478937995 on SSC12, is an interesting candidate gene for colour (a*, b* and score), firmness and marbling. MYH is a large family of motor proteins that share various common features such as ATP hydrolysis, actin binding and potential for kinetic energy transduction. A missense mutation in *MYH3* that induces an F4371 amino acid substitution, is known to segregate with distal arthrogryposis in a human family [44]. On SSC7, *ELOVL2* at 233 kb downstream of the significant SNP ss478937524 can be regarded as another promising candidate gene for LM_Firmness, because it is involved in the metabolism of lipids and lipoproteins in mammals [45]. The *KIF18A* gene, which is positioned 71 kb away from the significant SNP ss131184920 on SSC2, may contribute to the LM_Firmness trait measured in DLY pigs. KIF18A is a member of the kinesin superfamily of microtubule-associated molecular motors that use the energy derived from ATP hydrolysis to produce force and movement along the microtubule [46,47].

Based on the Erhualian dataset, four QTL that are involved in LM_MoistureContent were detected on SSC2, SSC8, SSC12 and SSC16. The significant SNP ss131186989 on SSC2 is just 2.5 kb downstream from the site of the *LDHA* gene, which encodes lactate dehydrogenase A that catalyzes the conversion of L-lactate and NAD to pyruvate and NADH in the final step of anaerobic glycolysis. LDHA is found predominantly in muscle tissue and belongs to the lactate dehydrogenase family [48]. On SSC16, the peak SNP ss131538960 was located between *MFAP3* (*microfibrillar-associated protein 3*) and *GALNT10* (*polypeptide N-acetylgalactosaminyltransferase 10*), within a region of 130 kb. *GALNT10* encodes a protein that may have increased catalytic activity towards glycosylated peptides compared to that towards non-glycosylated peptides [49], and, thus, it may be a better candidate gene than *MFAP3*. However, no obvious candidate genes were found for the other two loci on SSC8 and SSC12, where the nearest genes (*ENSSSCG00000029791* and *NFE2L1*) have no clear functional annotations correlated with the phenotype.

The most significant SNP ss131398982 for LM_ColorScore_36h in the DLY dataset is located only 47 kb away from the *ASNS* gene, which encodes a protein that is involved in the synthesis of asparagine. This gene complements a mutation in the temperature-sensitive hamster mutant ts11, which blocks progression through the G1 phase of the cell cycle at non-permissive temperatures [50]. Within the region between 127.41 and 134.39 Mb on SSC15, the effects of the QTL on LM_ColorM_a36h and LM_ColorScore_36h observed for the DLY population may be partially or completely due to mutations in the *PRKAG*3 gene at 133.80 Mb [51,52]. To confirm this, further analyses combining data based on 60K SNPs and *PRKAG3* sequence data are required.

Because precision of QTL location depends on various factors (e.g. marker density, population size, LD structure and precision of phenotypic measurement), it is necessary to be cautious when interpreting the results for loci with 95% confidence intervals that are larger than 1 Mb. One cannot exclude that the genes that underlie the QTL are outside the 1 Mb region that was investigated for candidate genes.

## Conclusions

In conclusion, we performed GWAS analyses for meat quality traits in Chinese Erhualian and western DLY populations. Overall, 28 and nine SNPs surpassed the significance level in the Erhualian and DLY pig data, respectively. The strongest association was obtained between drip loss and a SNP on SSC4 for the Erhualian population. Comparison of the loci that were detected by GWAS of Erhualian and DLY pig data demonstrated that only a few QTL were common to both pig lines, which reflects the large differences in genetic architecture of meat quality traits between the Chinese and western breeds. Our results suggest that several QTL affect multiple traits among which some are reported for the first time. The meta-analysis that combined GWAS of four populations uncovered seven novel loci, which were not detected by single-population GWAS. These novel loci represent variants that may be shared between these populations. For each of the QTL regions detected, we identified several candidate genes based on the possible relationship between gene function and the corresponding trait. This study provides new insights into the genetic basis of meat quality traits in pigs and further investigations are needed to guide the application of these QTL in future swine breeding programs.

## Additional files

Additional file 1: Figure S1. Quantile-quantile (Q-Q) plots of SNP distribution after quality control in GWAS for all tested meat quality traits. (A) Results for the Erhualian (EHL) population. (B) Results for the DLY population. Description: Datasets include 35 985 and 56 216 SNPs for the Erhualian and DLY populations, respectively. In the Q-Q plots, −log_10_ *P* values of observed association statistics on the Y-axis were compared to those of the association statistics expected under the hypothesis of no association on the X-axis. The solid line represents concordance between observed and expected values. The shaded region shows the 95% confidence interval based on Beta distribution. Genomic inflation factor, λ, is shown for each dataset. Additional file 2: Figure S2. Quantile-quantile (Q-Q) plots of SNP distribution after quality control in the GWAS meta-analysis. Description: The dataset includes 29 516 SNPs that are common among the four pig populations: Erhualian, DLY, F_2_ and Sutai. In the Q-Q plots, −log_10_ *P* values of observed association statistics on the Y-axis were compared to those of the association statistics expected under the hypothesis of no association on the X-axis. The solid line represents concordance between observed and expected values. The shaded region shows the 95% confidence interval based on Beta distribution. Genomic inflation factor, λ, is shown for each dataset. Additional file 3: Figure S3. Manhattan plots of GWAS for meat quality traits on LM and SM from Chinese Erhualian pigs and on LM from Western DLY pigs. (A) Results for the Erhualian population. (B) Results for the DLY population. LM, *longissimus* muscle; SM: *semimembranosus* muscle. Description: In the plots, negative log_10_ *P* values of the quantified SNPs were plotted against their genomic positions. SNPs on different chromosomes are indicated by different colours. Dotted, dashed and solid lines correspond to the thresholds of: (A) 1.00E-04, 2.77E-05 and 1.38E-06, respectively; (B) 1.00E-04, 1.78E-05 and 8.89E-07, respectively. Additional file 4: Figure S4. Manhattan plots of the GWAS meta-analysis for seven meat quality traits across four pig populations: Erhualian, DLY, White Duroc × Erhualian F_2_ and Sutai. Description: In these plots, negative log_10_ *P* values of the quantified SNPs were plotted against their genomic positions. SNPs on different chromosomes are indicated by different colours. Dotted, dashed and solid lines correspond to the thresholds of 1.00E-04, 3.39E-05 and 1.69E-06, respectively.
