# Supplementary figures and images for: Genome-wide association analyses for meat quality traits in Chinese Erhualian pigs and a Western Duroc × (Landrace × Yorkshire) commercial population
Source: Genet Sel Evol. 2015 May 12;47(1):44. doi: 10.1186/s12711-015-0120-x (PMC4427942; doi:10.1186/s12711-015-0120-x)

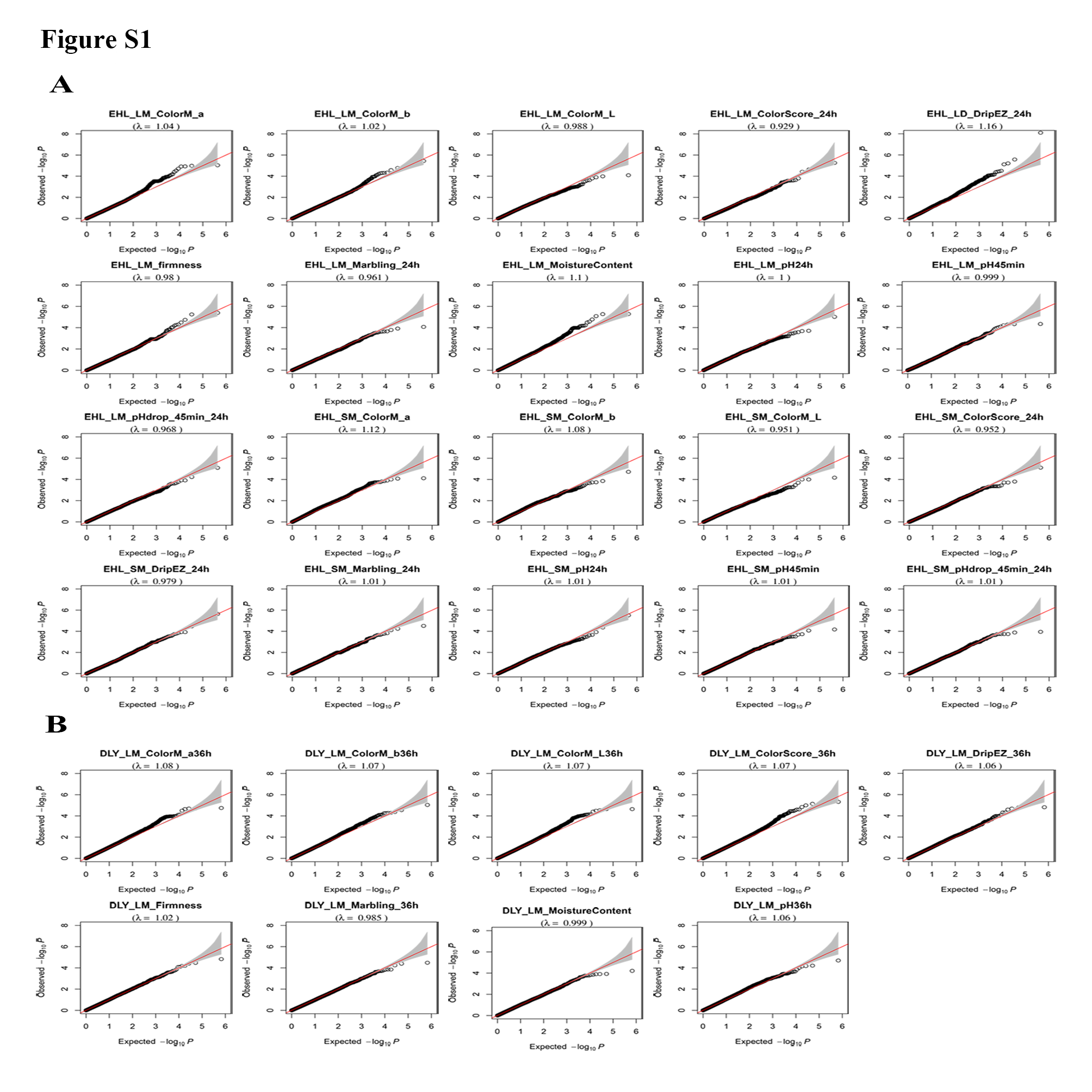

Supplement: Additional file 1: Figure S1. — Quantile-quantile (Q-Q) plots of SNP distribution after quality control in GWAS for all tested meat quality traits. (A) Results for the Erhualian (EHL) population. (B) Results for the DLY population. Description: Datasets include 35 985 and 56 216 SNPs for the Erhualian and DLY populations, respectively. In the Q-Q plots, −log10 P values of observed association statistics on the Y-axis were compared to those of the association statistics expected under the hypothesis of no association on the X-axis. The solid line represents concordance between observed and expected values. The shaded region shows the 95% confidence interval based on Beta distribution. Genomic inflation factor, λ, is shown for each dataset. [file 12711_2015_120_MOESM1_ESM.tiff]

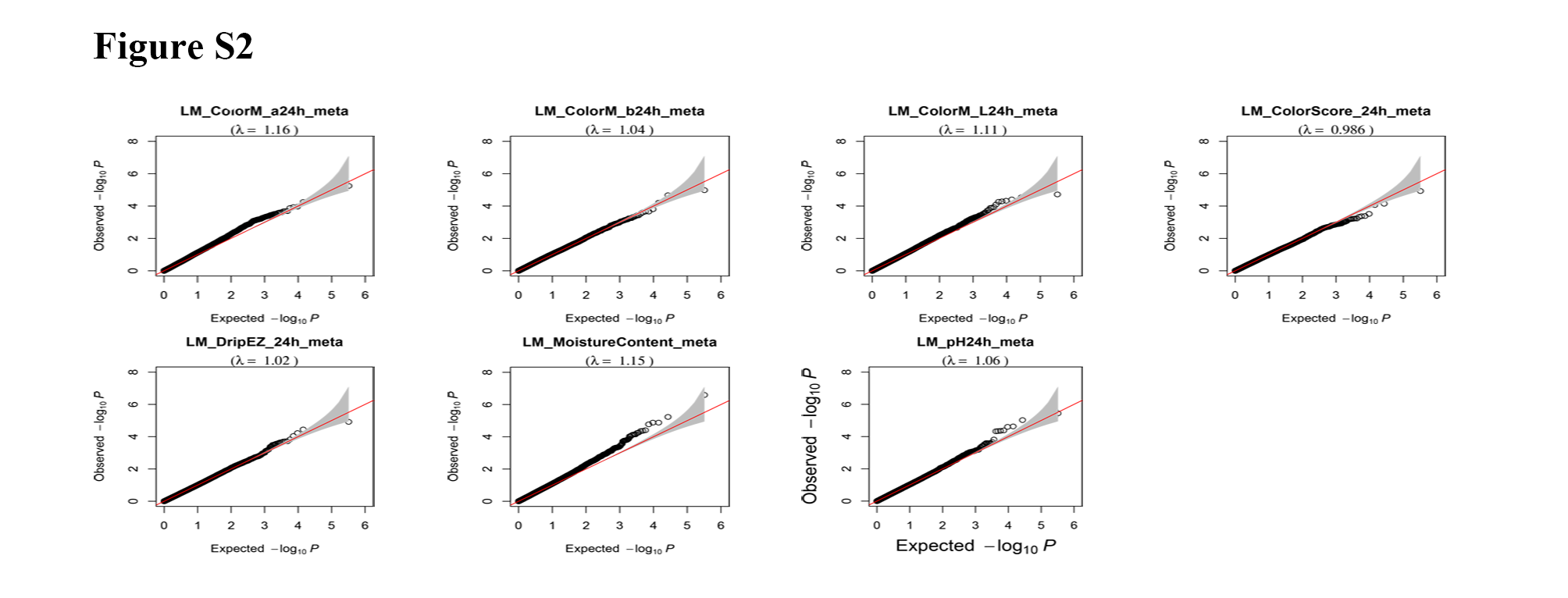

Supplement: Additional file 2: Figure S2. — Quantile-quantile (Q-Q) plots of SNP distribution after quality control in the GWAS meta-analysis. Description: The dataset includes 29 516 SNPs that are common among the four pig populations: Erhualian, DLY, F2 and Sutai. In the Q-Q plots, −log10 P values of observed association statistics on the Y-axis were compared to those of the association statistics expected under the hypothesis of no association on the X-axis. The solid line represents concordance between observed and expected values. The shaded region shows the 95% confidence interval based on Beta distribution. Genomic inflation factor, λ, is shown for each dataset. [file 12711_2015_120_MOESM2_ESM.tiff]

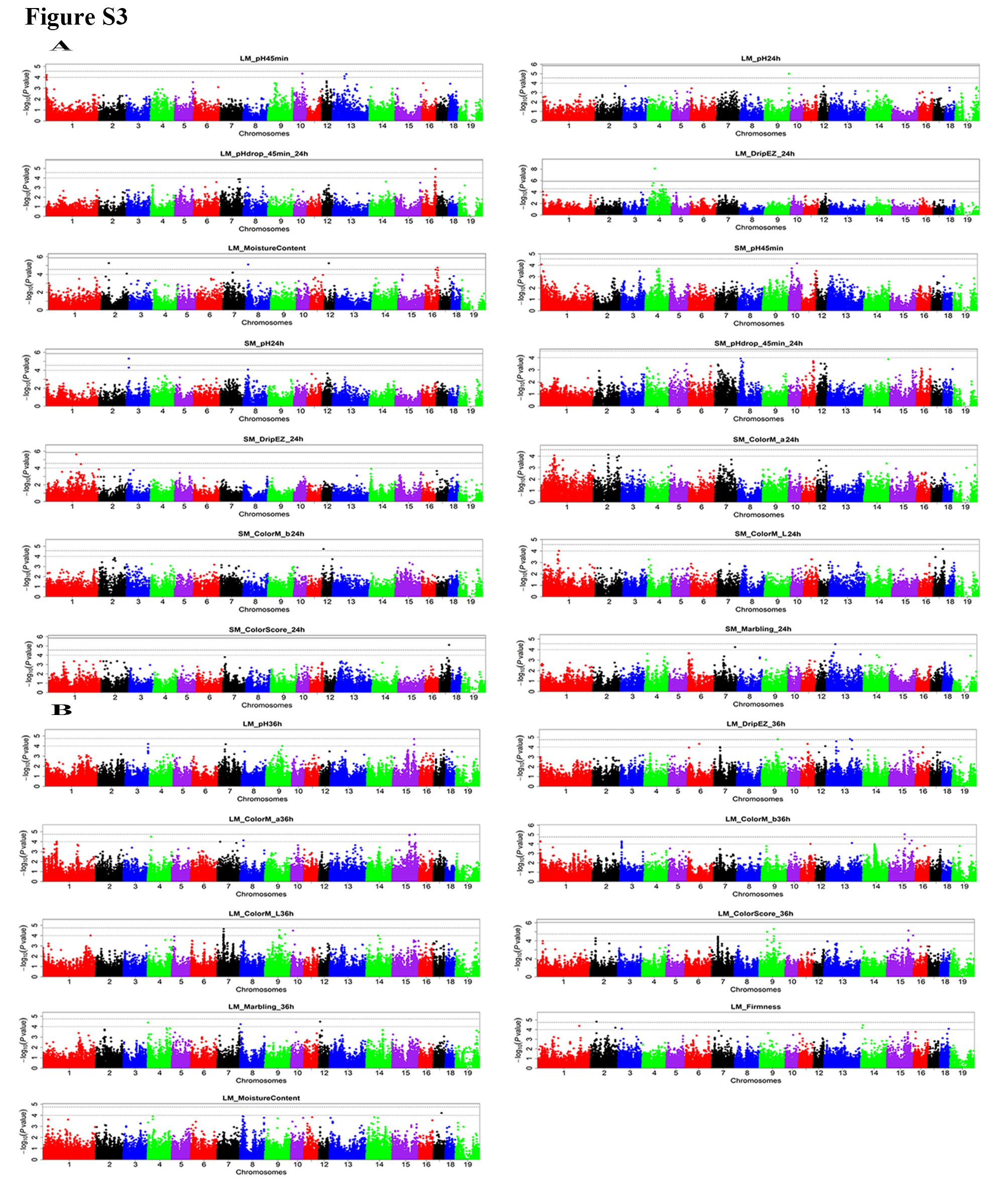

Supplement: Additional file 3: Figure S3. — Manhattan plots of GWAS for meat quality traits on LM and SM from Chinese Erhualian pigs and on LM from Western DLY pigs. (A) Results for the Erhualian population. (B) Results for the DLY population. LM, longissimus muscle; SM: semimembranosus muscle. Description: In the plots, negative log10 P values of the quantified SNPs were plotted against their genomic positions. SNPs on different chromosomes are indicated by different colours. Dotted, dashed and solid lines correspond to the thresholds of: (A) 1.00E-04, 2.77E-05 and 1.38E-06, respectively; (B) 1.00E-04, 1.78E-05 and 8.89E-07, respectively. [file 12711_2015_120_MOESM3_ESM.tiff]

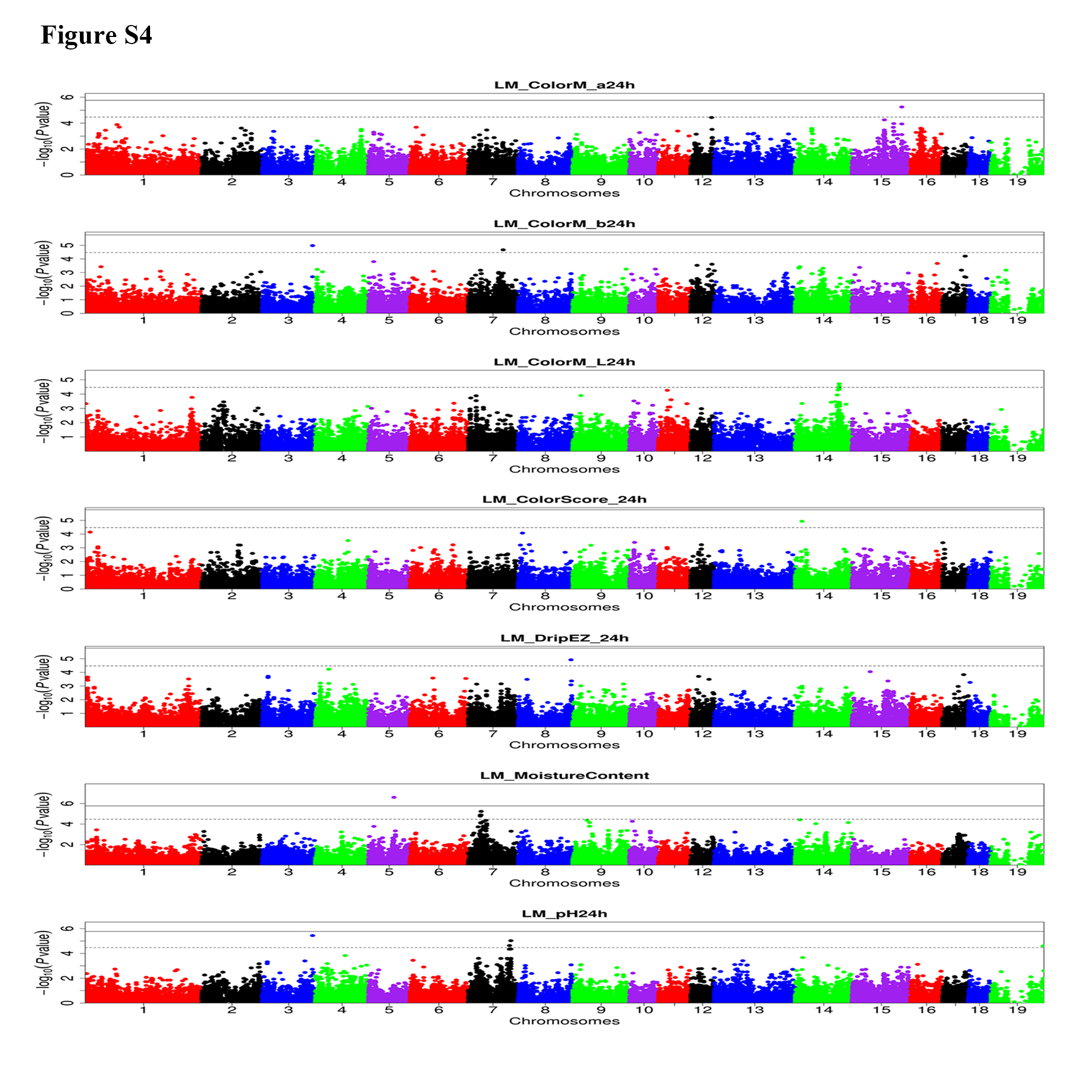

Supplement: Additional file 4: Figure S4. — Manhattan plots of the GWAS meta-analysis for seven meat quality traits across four pig populations: Erhualian, DLY, White Duroc × Erhualian F2 and Sutai. Description: In these plots, negative log10 P values of the quantified SNPs were plotted against their genomic positions. SNPs on different chromosomes are indicated by different colours. Dotted, dashed and solid lines correspond to the thresholds of 1.00E-04, 3.39E-05 and 1.69E-06, respectively. [file 12711_2015_120_MOESM4_ESM.tiff]
